# Safety Evaluation of Oral Care Probiotics *Weissella cibaria* CMU and CMS1 by Phenotypic and Genotypic Analysis

**DOI:** 10.3390/ijms20112693

**Published:** 2019-05-31

**Authors:** Mi-Sun Kang, Ji-Eun Yeu, Sang-Phil Hong

**Affiliations:** 1 Research Institute, Oradentics Inc., 1805-ho, 25 Seongsuil-ro-4-gil, Seongdong-gu, Seoul 04781, Korea; ji-eun85@oradentics.com; 2 Department of Food and Nutrition, Hanyang University, 222 Wangsimni-ro, Seongdong-gu, Seoul 04763, Korea; 3 Division of Strategic Food Research, Korea Food Research Institute (KFRI), 245, Nongsaengmyeong-ro, Iseo-myeon, Wanju-gun, Jeollabuk-do 55365, Korea; sphong@kfri.re.kr

**Keywords:** safety, oral care, probiotics, *Weissella cibaria*, antibiotic resistance

## Abstract

*Weissella cibaria* CMU and CMS1 are known to exert beneficial effects on the oral cavity but have not yet been determined to be generally recognized as safe (GRAS), although they are used as commercial strains in Korea. We aimed to verify the safety of *W. cibaria* CMU and CMS1 strains through phenotypic and genotypic analyses. Their safety was evaluated by a minimum inhibitory concentration assay for 14 antibiotics, DNA analysis for 28 antibiotic resistance genes (ARGs) and one conjugative element, antibiotic resistance gene transferability, virulence gene analysis, hemolysis, mucin degradation, toxic metabolite production, and platelet aggregation reaction. *W. cibaria* CMU showed higher kanamycin resistance than the European Food Safety Authority (EFSA) cut-off, but this resistance was not transferred to the recipient strain. *W. cibaria* CMU and CMS1 lacked ARGs in chromosomes and plasmids, and genetic analysis confirmed that antibiotic resistance of kanamycin was an intrinsic characteristic of *W. cibaria*. Additionally, these strains did not harbor virulence genes associated with pathogenic bacteria and lacked toxic metabolite production, β-hemolysis, mucin degradation, bile salt deconjugation, β-glucuronidase, nitroreductase activity, gelatin liquefaction, phenylalanine degradation, and platelet aggregation. Our findings demonstrate that *W. cibaria* CMU and CMS1 can achieve the GRAS status in future.

## 1. Introduction

“Probiotics” is a compound word consisting of “pro” (benign) and “biotics” (biologically relevant), which refers to living bacteria that enter the body and exert a beneficial effect [1]. The product market that utilizes probiotics is steadily increasing. Lactic acid bacteria (LAB) have been used as the main probiotic ingredients, and living microorganisms are becoming an important requirement for the concept of probiotics [2]. Probiotic products have been consumed as fermented milk products prepared using LAB such as *Lactobacillus* and *Bifidobacterium*, but they have also recently been sold in the form of powders or tablets [3]. With the growth of the market, the past two decades have witnessed steady progress in research on the potential effects of probiotics on the treatment and prevention of diseases, and several *Bifidobacterium* and *Lactobacillus* species of probiotics have been developed and marketed [4]. These types of probiotics are generally recognized as safe (GRAS) because they show long-term safety in fermented foods or dairy products. However, the United Nations Food and Agriculture Organization (FAO)/World Health Organization (WHO) consider it important to conduct a minimum safety assessment including antibiotic resistance, specific metabolite production such as d-lactate and ammonia, side effects in humans, toxin production, and potential hemolysis, even for microbial populations classified as GRAS [5].

Over the last few years, the remarkable advancement of molecular biology has made it possible to study oral microorganisms at unprecedented levels. It is known that 700–1000 different microorganisms are found in the oral cavity depending on the bacterial habitat such as the tongue, teeth, gums, buccal mucosa, palate, and tonsil [6]. Many researchers are trying to clarify the evidence supporting the oral health benefits of probiotics through clinical studies. In numerous studies, probiotics have been investigated and commercialized for the purpose of preventing or treating oral diseases such as dental caries and periodontal disease associated with changes in microbial composition, since probiotics are likely to modify oral microorganisms [7,8,9]. Therefore, when selecting suitable probiotic species, the normal oral habitat and its association with health should be considered. Because criteria for selection of probiotics are not based on a microbiology classification system, there are no specific and clear selection criteria; selection is rather based on the results of FAO/WHO guidelines or findings of microbiologists. Selection criteria are classified into safety, productivity, and functional aspects. The source of the probiotic candidate strains is important, and probiotic strains isolated from the human body should be used when applied to the human body [5]. Since the effect of probiotics depends on the strain, it is necessary to verify the identity, characterization, and nonpathogenicity of the genotype or phenotype for safety and human use.

Several specific strains of *Lactobacillus brevis*, *Lactobacillus reuteri*, *Lactobacillus salivarius*, *Streptococcus salivarius*, and *Weissella cibaria* are commercially available oral care probiotics, all of which are microorganisms isolated from the oral cavity [10,11]. *W. cibaria* is a Gram-positive lactic acid bacterium that is non-spore-forming, nonmotile, heterofermentative, catalase-negative, and short rod-shaped. *W. cibaria* CMU (oraCMU) and CMS1 (oraCMS1) were isolated from saliva samples of 460 kindergarten children who had little supragingival plaque and no oral diseases including dental caries [12,13,14]. These *W. cibaria* strains have been shown to exert inhibitory effects on dental caries, halitosis, and periodontal disease through in vitro experiments, in vivo experiments, and clinical studies [12,13,14]. Recent studies have also shown that the antimicrobial activity of *W. cibaria* CMU is due to at least three inhibitory mechanisms towards the growth of periodontal bacteria: acidic pH, hydroxyl radicals, and the secretion of specific proteins [15]. These strains have been registered as safe raw materials by the Korea Food and Drug Administration (KFDA) [16] and are actively commercialized as oral care probiotics in Korea.

In addition to animal and clinical trials, the safety of *W. cibaria* CMU and CMS1 has been verified by the absence of adverse reactions to ingestion of the product in recent years, but additional safety data are required for them to achieve the GRAS status. Therefore, the purpose of this study was to verify the safety of *W. cibaria* CMU and CMS1 through experiments and other safety studies recommended by FAO/WHO.

## 2. Results

### 2.1. Antibiotic Resistance

The antibiotic susceptibility of two *W. cibaria* strains, three commercial probiotic isolates, and *Enterococcus faecalis* ATCC 29,212 was analyzed, and the strains were classified as either resistant or sensitive based on the cut-off values from the European Food Safety Authority (EFSA) 2012 guidelines [17]. The minimum inhibitory concentration (MIC) values of all *W. cibaria* CMU, with the exception of kanamycin, were equal to or lower than the established EFSA cut-off values of *Lactobacillus* obligate heterofermentative species (Table 1). The MIC values of other commercial probiotics including *L. reuteri* and *L. salivarius* were also found to be equal to or lower than the EFSA cut-off values, except for kanamycin, whereas *E. faecalis* was found to be resistant to gentamicin, streptomycin, clindamycin, and tetracycline.

### 2.2. PCR Detection for Antibiotic Resistance Genes (ARGs)

PCR analyses for twenty-eight antibiotic resistance genes (ARGs) including gentamicin (*aac*(6)-*aph*(2)), kanamycin (*aadA*, *ant*(2”)-*I*, *aph*(3”)-*I*, and *aph*(3”)-*III*), streptomycin (*aadE*, *ant6*), tetracycline (*tet*(M), *tet*(K), and *tet*(W)), erythromycin (*erm*(B), *erm*(B)*-1*, and *erm*(C)), clindamycin (*lnu*(A), *lnu*(B)), chloramphenicol (*catA*, *cat*), ampicillin (*blaZ*, *bla*, and *mecA*), vancomycin (*vanE*, *vanX*), quinupristin/dalfopristin (*vatC*, *vatE*), linezolid (*cfr*), rifampicin (*rpoB*), and ciprofloxacin (*gyrA*, *parC*), as well as one conjugative transposon integron (Tn*916*/Tn*1545*) on chromosomal or plasmid DNA, were conducted (Table 2). There were no ARGs in the chromosome and plasmid DNA of *W. cibaria* strains or commercial probiotics. However, the tetracycline-resistance gene *tet*(M) and conjugative transposon integron-specific genes in chromosomes and plasmid DNA of the *E. faecalis* strain showed 90% and 99% homology with the *Clostridium difficile* 630 *tet*(M) and *Streptococcus suis* integrative and conjugative elements, respectively.

### 2.3. Transferability of ARGs

The transferability of kanamycin resistance of the CMU strain was tested using *Lactobacillus rhamnosus*, a recipient strain that is susceptible to kanamycin. In order to test the transferability of vancomycin resistance of CMU, *E. faecalis* was used as the recipient strain because of its high vancomycin sensitivity. As shown in Table 3, no colonies of presumptive transconjugants were found from the selective agar plates after mating.

### 2.4. Genome Analysis for ARGs

A total of 7828 ARGs were used in the analysis by the Antibiotic Resistance Genes Database (ARDB) tool, and an *E* value of 1 × 10^−10^ was used as the cutoff reference value. Genome analysis results showed that no genes in *W. cibaria* strains were homologous (% identical) at the chromosome and plasmid levels. In addition, no ARGs were found from *W. cibaria* genome data in the Resistance Gene Identifier (RGI) results (Table 4).

### 2.5. Virulence Genes

Genomic sequences of *W. cibaria* CMU and CMS1 were compared with genomic sequences of four well-known pathogens (*Escherichia coli*, *Listeria*, *Staphylococcus aureus*, and *Enterococcus*). Virulence genes including the Shiga toxin gene and virulence genes for *E. coli*; virulence genes for *Listeria*; and exoenzyme genes, host immune alteration or evasion genes, and toxin genes for *S. aureus* were included. No virulence genes were shown in the genomic sequences of *W. cibaria* strains or those of *L. rhamnosus*. On the other hand, in the case of *E. faecalis*, 13 virulence genes were identified (Table 5).

### 2.6. Hemolytic Properties

**Figure 1:**
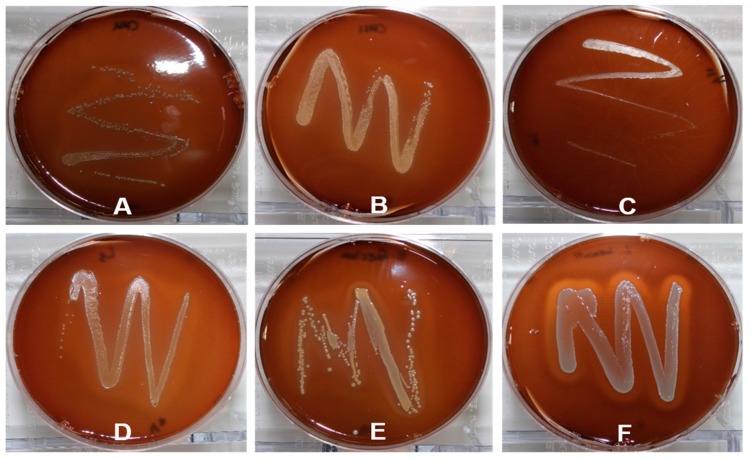
Hemolysis activity of *W. cibaria* strains and commercial probiotics including *L. reuteri* and *L. salivarius*. The plates inoculated with *L. ivanovii* KCTC 3444 as a positive control demonstrated β-hemolysis. (**A**) *W. cibaria* CMU; (**B**) *W. cibaria* CMS1; (**C**) *L. reuteri*; (**D**) *L. salivarius*; (**E**) *E. faecium*; and (**F**) *L. ivanovii.*

The hemolytic activity of *W. cibaria* strains was assessed using 5% sheep blood agar. Colonies of *W. cibaria* and commercial probiotic strains did not show hemolysis, whereas colonies of *Lactobacillus ivanovii* demonstrated hemolysis (Figure 1).

### 2.7. Mucin Degradation

**Figure 2:**
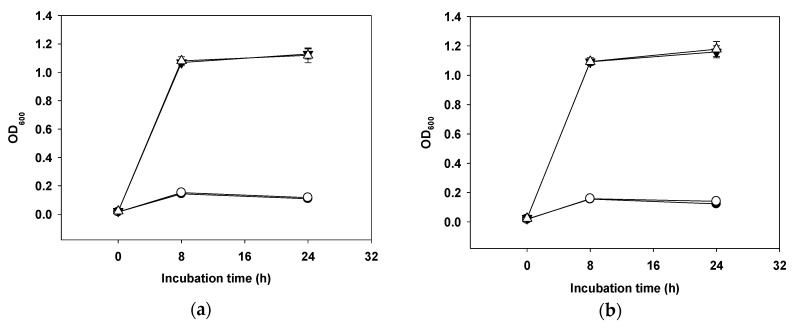

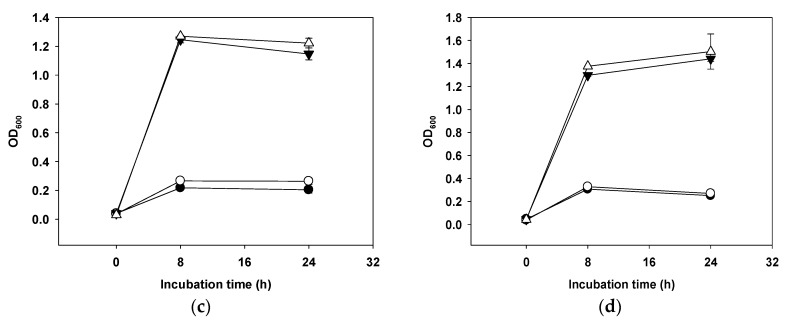
Growth curves for *W. cibaria* CMU (**a**), *W. cibaria* CMS1 (**b**), *L. reuteri* (**c**), and *L. salivarius* (**d**) in modified MRS medium with various carbon sources. Basal medium (glucose-free) (●), basal medium with 0.3% mucin (○), basal medium with 1.0% glucose (▼), and basal medium with 0.3% mucin and 1.0% glucose (△).

The mucolytic capacity of *W. cibaria* was measured using various modified media including basal medium (glucose-free De Man, Rogosa, and Sharpe broth (MRS broth)), basal medium with 0.3% mucin, 1.0% glucose, or 1.0% glucose with 0.3% mucin. The cell growth rates were examined by measuring optical density at 600 nm (OD_600_) and pH changes in culture. As shown in Figure 2, *W. cibaria* strains and commercial probiotic strains were unable to grow in medium containing mucin.

### 2.8. Toxic Metabolite Production

**Figure 3:**
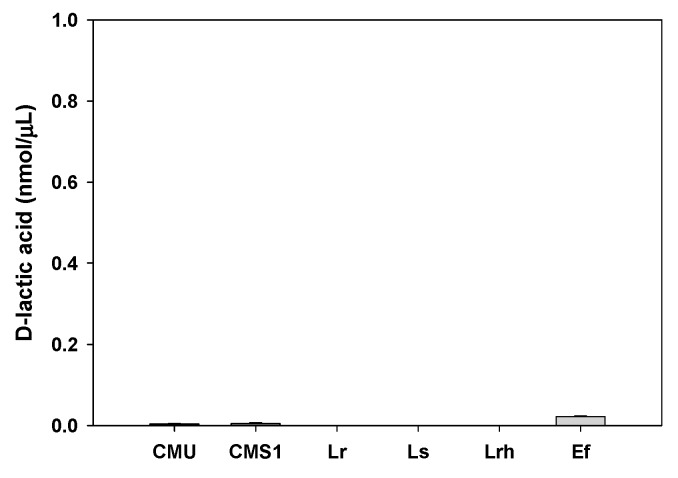
d-lactic acid production by the studied bacterial strains. CMU, *W. cibaria* CMU; CMS1, *W. cibaria* CMS1; Lr, *L. reuteri*; Ls, *L. salivarius*; Lrh, *L. rhamnosus*; and Ef, *E. faecium* ATCC 19434.

**Figure 4:**
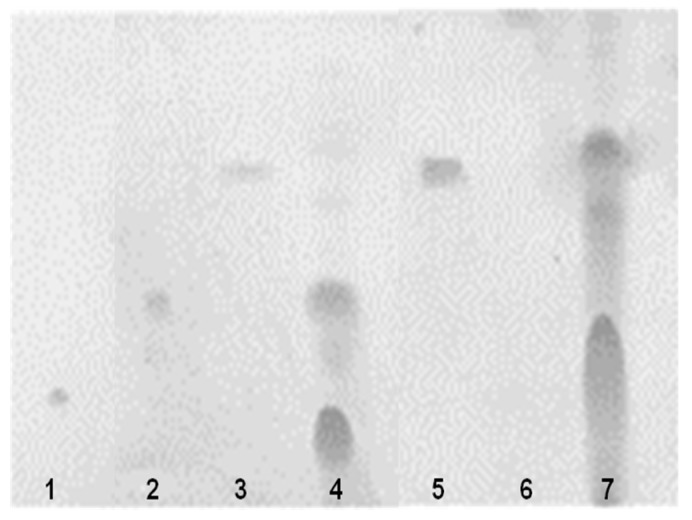
Thin-layer chromatogram of the deconjugation products of bile salts by *W. cibaria* CMU. 1, cholic acid; 2, chenodeoxycholic acid; 3 and 5, deoxycholic acid; 4, *W. cibaria* CMU; 6, lithocholic acid; and 7, *E. limosum* ATCC 8486.

**Figure 5:**
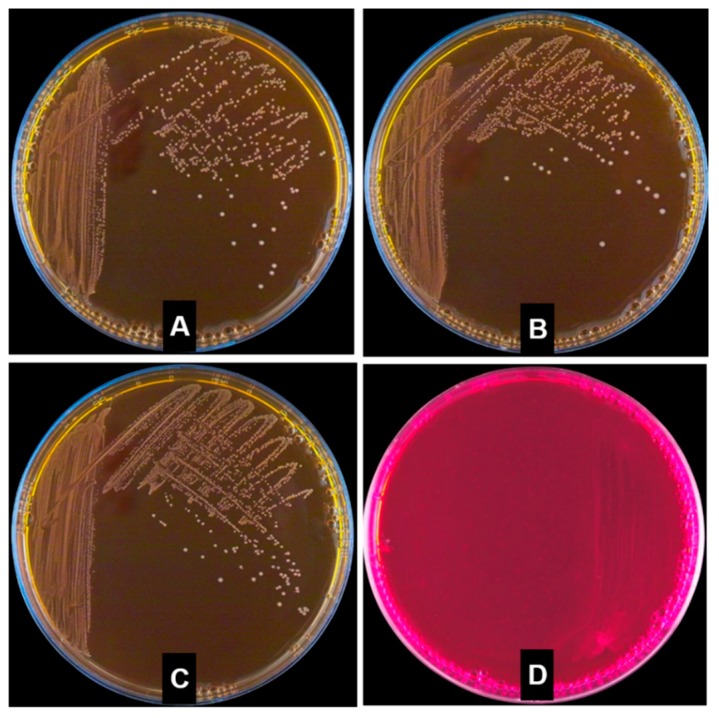
Ammonia production by *W. cibaria* strains. (**A**) *W. cibaria* CMU; (**B**) *W. cibaria* CMS1; (**C**) *W. cibaria* KCTC 3807; and (**D**) *P. vulgaris* KCTC 2512.

**Figure 6:**
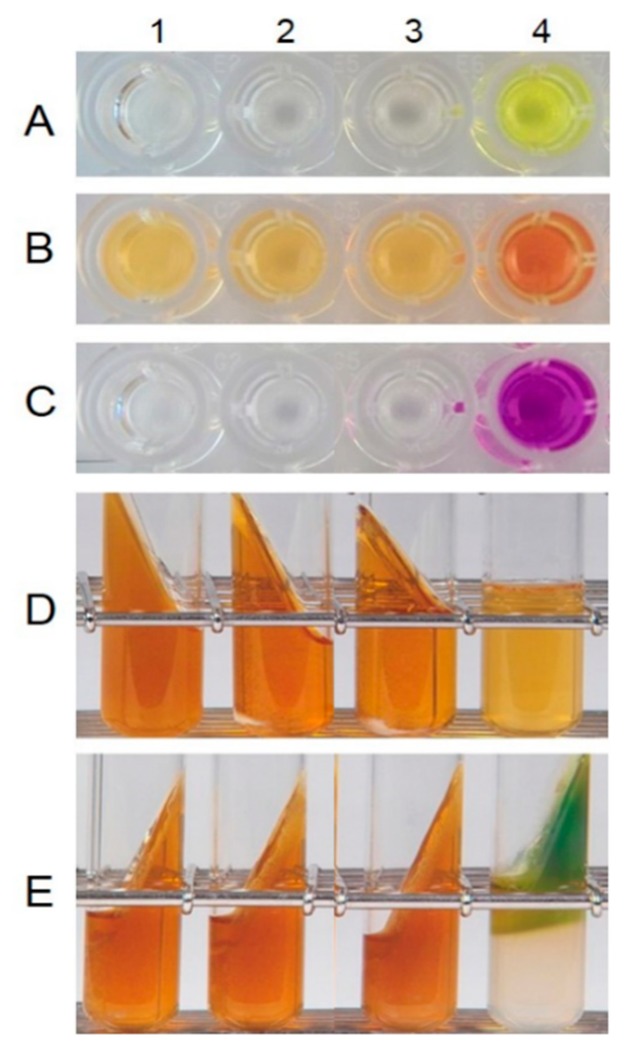
The β-glucuronidase activity (**A**), indole production (**B**), nitroreductase activity (**C**), gelatin liquefaction (**D**), and phenylalanine degradation (**E**) by *W. cibaria* strains. 1, *W. cibaria* CMU; 2, *W. cibaria* CMS1; 3, *W. cibaria* KCTC 3807; and 4, positive control (from **A** to **C**, *E. coli* O157:H7; **D**, *P. aeruginosa* KCCM 11328; and **E**, *P. vulgaris* KCTC 2512).

The production of d-lactic acid by *W. cibaria* was determined by enzymatic assays involving d-lactate dehydrogenase. As shown in Figure 3, *W. cibaria* strains did not produce d-lactic acid in a manner similar to commercial probiotic strains. The analysis of bile salt deconjugation by HPLC demonstrated that *W. cibaria* hydrolyzed the conjugated bile acids taurocholic acid (TCA) and glycocholic acid (GCA) by 96.97% and 98.14%, respectively. Additionally, through thin-layer chromatographic (TLC) analysis, we found that *W. cibaria* was not capable of converting cholic acid (CA) and chenodeoxycholic acid (CDCA), the primary bile acids, to deoxycholic acid (DCA) and lithocholic acid (LCA), the secondary bile acids. On the other hand, CA and CDCA were converted to DCA and LCA, respectively, by *Enterococcus limosum*, used as a positive control (Figure 4). As a result of the ammonia production, *W. cibaria* did not show any urease activity, whereas *Pseudomonas vulgaris* (used as a positive control) produced ammonia (Figure 5). *W. cibaria* did not show β-glucuronidase activity, whereas *E. coli* as a positive control changed the color of the media (Figure 6A). In the indole production test, *W. cibaria* did not cause a change in color, but *E. coli*, which is an indole producer, showed a red color (Figure 6B). *W. cibaria* also did not show nitroreductase activity, in contrast to the positive strain *E. coli*, which changed the color of the media to violet (Figure 6C). Proteinase activity of *W. cibaria* was assessed with a tube containing nutrient gelatin. The gelatin agar media inoculated with *W. cibaria* remained solid, whereas the proteinase-positive species *Pseudomonas aeruginosa* was shown to liquefy the gelatin (Figure 6D). *W. cibaria* strains did not show any color change in phenylalanine slant agar, whereas the positive strain *P. vulgaris* caused the media to turn green (Figure 6E). In the API ZYM test to detect the bacterial enzyme activities, *W. cibaria* strains showed negative results, except for two enzymatic reactions (acid phosphatase and naphthol-AS-BI-phosphohydrolase). There was a difference in the enzymatic profile between the commercial probiotics (Table 6).

### 2.9. Platelet Aggregation

**Figure 7:**
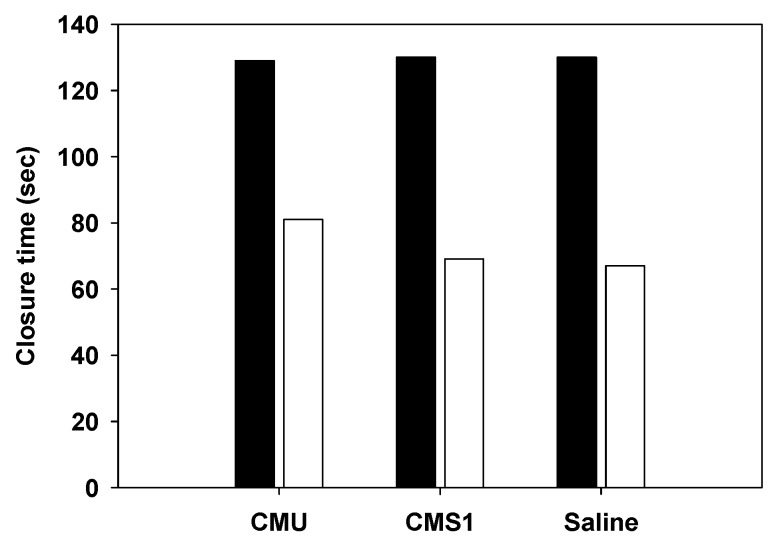
Platelet aggregation by *W. cibaria* strains. CMU, *W. cibaria* CMU; CMS1, *W. cibaria* CMS1. Saline was used as a negative control. C/EPI (■); C/ADP (□).

The potential of *W. cibaria* for platelet aggregation was evaluated in whole blood specimens using a platelet function analyzer. As shown in Figure 7, the closure time was similar in both saline and *W. cibaria* strains, indicating that *W. cibaria* CMU and CMS1 strains did not cause platelet aggregation.

## 3. Discussion

In order to promote market growth and consumer acceptance for probiotics, their safety and functionality should be evaluated using a global standard. The use of probiotics is considered a good alternative to antibiotics because antibiotic abuse is associated with adverse events such as the emergence of super bacteria. Recently, human–microbial interactions and interactions between microbial genes and diseases have been studied, and new markets for disease treatments using probiotics beyond food and dairy products are expected to be pioneered [4]. Meanwhile, mutation of specific genes among microorganisms can facilitate the appearance of super bacteria with antibiotic resistance [18]. Traditionally, antibiotic resistance plasmids such as *erm* and *tet* vectors have been found in *Lactobacillus* and *Bifidobacterium* [19]. Lactic acid bacteria (LAB) themselves have been found to be resistant to ARGs because they can function as donors of antibiotic resistant plasmids [19].

As the number of pathogens with antibiotic resistance has increased, LAB have been shown to deliver antibiotic resistance to pathogens [19]. In the case of probiotics, strains with strong antibiotic resistance have been used, and antibiotic resistance can cause serious problems when transferred to other intestinal bacteria, especially pathogenic bacteria. As a result of the safety issues associated with LAB, the antibiotic resistance of *W. cibaria* strains were compared with that of other commercial LAB in this study. In relation to *Weissella* spp., studies on the cut-off values have not been defined by EFSA [17]. Therefore, an antibiotic susceptibility test of *W. cibaria* was performed to determine the strain corresponding to a *Lactobacillus* obligate heterofermentative strain, based on the 2012 EFSA cut-off value, which reflects the characteristics of the strain. As a result, *W. cibaria* CMU as well as commercially available LAB including *L. reuteri* and *L. salivarius* were found to be equal to or lower than the 2012 EFSA cut-off value, except for kanamycin. The resistance tendency of *W. cibaria* strains to kanamycin was similar to that reported in previous studies [20,21]. For vancomycin, it is known that the facultative and obligate fermentative *Lactobacillus* groups are intrinsically resistant [17]. In this context, all tested *Lactobacillus* spp. and *W. cibaria* strains showed MICs ≥ 256 mg/L for vancomycin, suggesting that vancomycin resistance could be considered as intrinsic.

In the present study, antibiotic resistance transferability studies were conducted to confirm the nature of this resistance. Since *W. cibaria* CMU showed high antibiotic resistance to vancomycin and kanamycin in these antimicrobial susceptibility tests, *W. cibaria* CMU was conjugally mated with *E. faecalis* ATCC 29212 and *L. rhamnosus* GG by filter mating. No colonies of presumptive vancomycin or kanamycin transconjugants were found on the selective plates after mating. Thus, this study demonstrated that the antibiotic resistance of *W. cibaria* CMU was not transferred to the recipient strains.

In addition, the presence of ARGs on chromosomes and plasmids of *W. cibaria* strains was confirmed by PCR analysis and a Comprehensive Antibiotic Resistance Database Basic Local Alignment Search Tool (CARD BLAST) search in this study [22]. The amplicons for ARGs were not identified in the CARD BLAST search. A conjugative transposon integron-specific gene was also analyzed and showed no homology. This confirmed that *W. cibaria* strains did not have ARGs in either chromosomal or plasmid DNA. In our study, we detected that *E. faecalis* ATCC 29212 had a tetracycline-resistance gene (*tet*(M)) and also found a conjugative element, Tn*916*/Tn*1545*, which is known to carry the tetracycline resistance gene [23].

A number of studies have been conducted on antibiotic resistance to LAB over the last 10 years, and *Enterococcus* is no longer GRAS [24,25]. Enterococci are known to be involved in fermentation and flavor fermentation of dairy products and have long been used as a fermentation seed and in probiotics [26]. However, they have a high antibiotic resistance gene acquisition ability and are frequently detected in hospital-acquired and opportunistic infections [27]. Although the incidence of enterococcal infection due to enterococci-containing probiotic products has not yet been reported, the safety of enterococci isolated from foods remains controversial because of their risk factors. Currently, they are classified as microorganisms requiring safety evaluation for use in food [28]. These antibiotic-resistance problems have been observed not only in enterococci but also in *Lactococcus*, *Lactobacillus*, *Leuconostoc*, *Pediococcus*, and *Bifidobacterium* and have been a serious risk factor in the field of food microbiology [19,20,21].

In general, the safety assessment requirements for LAB strains for GRAS recognition have been accompanied by studies to determine whether antibiotic profiles and antibiotic resistance can be transferred. In this study, ARGs were investigated using WGS analysis of *W. cibaria* strains, and very little homology was observed between the genomic sequences of the *W. cibaria* strains. These results indicated that there were no ARGs in *W. cibaria* strains. In addition to antibiotic resistance, safety evaluation of LAB is required to consider various other risk factors such as virulence genes, hemolysis, bile salt deconjugation, as well as d-lactate and ammonia production [17]. In this study, no virulence genes were found in the genomic sequences of *W. cibaria* strains. This result shows that the genomic sequences of *W. cibaria* strains do not include toxic or pathogenic genes related to *E. coli*, *Enterococcus*, *Listeria*, and *S. aureus*. Hemolysin is a toxin that damages the cell membrane of the host cell and destroys the cell, leading to death [29]. Hemolysis is a phenomenon that occurs when a red blood cell is destroyed, and the soluble components are eluted and become a red transparent liquid. Since β-hemolysis is associated with pathogenicity, the test bacteria were cultured in a blood agar medium to observe the hemolysis. In the present study, *W. cibaria* strains as well as commercial probiotics had no hemolysis activity.

The mucin layer of the gastrointestinal tract protects epithelial cells from digestive enzymes present in gastric juice and from shear and ingested pathogens produced by digestive processes [30]. Therefore, if the mucus layer is altered, the host may be damaged. Our results demonstrated that *W. cibaria* strains could not grow in medium containing mucin and could not induce mucolytic degradation in vitro.

The formation reaction of bile salts occurs by the action of microorganisms in the human colon [31]. The enzyme 7α-dehydroxylase produces the secondary bile salts deoxycholic acid and lithocholic acid by removing the 7α-dehyroxyl group from cholic acid and chenodeoxycholic acid, which are primary bile salts. Secondary bile acids are implicated as etiologic factors of colon cancer [31]. To date, the activity of 7α-dehydroxylase has been detected in some strains of *Eubacterium* and *Clostridium*, but it has been argued that 7α-dehydroxylase is also present in LAB such as *Bifidobacterim* and *Lactobacillus* [32]. In this study, we found that *W. cibaria* hydrolyzed taurocholic and glycocholic acid but had no ability to convert to secondary bile acid. This result agrees with that of a published report [33].

Lactic acid has two optical isomers, L and D, and human metabolites are generally L-lactic acid because only human L-lactate dehydrogenase (LDH) is present [34]. d-lactate, which is produced mainly by bacteria, can hardly be measured in the human body or only in a very small amount. However, the relationship between the elevation of d-lactic acid in blood and the functional impairment of the liver and kidney has been investigated [35]. Therefore, we compared the ability of *W. cibaria* strains to produce d-lactate with that of other LAB. Our results demonstrated that the *W. cibaria* strains used in this study could not produce d-lactic acid.

Some bacteria contain urease, which catalyzes the hydrolysis of urea and yields alkaline ammonia products, which turn the pH indicator in the urease agar to a red or pink color. As ammonia is produced, the medium turns bright pink or cerise when the pH rises to 8.1 or higher [36]. No studies of the urease activity of *W. cibaria* isolated from the oral cavity have been reported. In this work, none of the *W. cibaria* strains produced color, suggesting that *W. cibaria* did not produce urease.

β-glucuronidase is one of the major enzymes in the liver and has been associated with the generation of potential carcinogenic metabolites [37]. β-glucuronidase hydrolyzes the substrate *ρ*-nitrophenyl-β-d-glucuronide to produce nitrophenol. In this study, β-glucuronidase was not detected in any of the *W. cibaria* strains. The results of the enzymatic profiles of *W. cibaria* strains evaluated by API ZYM were also similar to a report by Muñoz-Atienza et al. [33]. When tryptophan is degraded by tryptophanase, indole is formed with pyruvic acid and ammonia [38]. It is thought that indole is not formed by body tissues but is produced by tryptophan by the action of bacteria in the intestines. In this study, we determined whether *W. cibaria* strains produced indole using Kovac’s reagent. The positive control, *E. coli*, showed a pink or red color in the top alcohol layer, but the *W. cibaria* strains did not produce any indole when there were no yellow color changes. Nitroreductase converts 4-nitrobenzoic acid to 4-aminobenzoic acid, and diazonium salt is produced by the diazotization of amine [39]. In this study, the positive control *E. coli* changed the color of media to red while the *W. cibaria* strains did not produce a red color, suggesting that they did not possess nitrate reductase. To our knowledge, this is the first report investigating the nitroreductase activity of *W. cibaria* and its ability to form indole.

Bacterial pathogenicity is often dependent on cell penetration ability, and cell invasion requires protein degradation ability [40]. In this study, we investigated the degradability of proteins by determining whether a gelatin liquefaction phenomenon existed. A positive reaction means that the proteolytic enzyme is secreted and the possibility of cell penetration is high. The results of gelatin resolutions for all three *W. cibaria* strains including the standard type strain were negative. In the case of *P. aeruginosa* with gelatin resolution, the gelatin was not hardened.

When amino acids are decomposed by carbonic acid enzymes, harmful amines are often formed. Examples are histamine produced from histidine, cadaverine produced from lysine, and agmatine produced from arginine. Some intestinal microbes such as *E. coli* and *Clostridium* produce phenolic compounds from tyrosine and phenylalanine, which have been reported to correlate with carcinogenesis [37]. In this study, the phenylalanine deamines of intestinal microorganisms were examined for the production of phenylpyruvic acid by using phenylalaninase to produce phenylpyruvic acid, which turned green when it reacted with 10% ferric chloride. In this study, the positive strain *P. vulgaris* showed a dark green color that was produced by phenylpyruvic acid and 10% ferric chloride. However, the *W. cibaria* strains did not show any color change, suggesting that *W. cibaria* did not produce phenylpyruvic acid.

Intestinal bacteria such as LAB can enter the blood vessels. This can be attributed to unsanitary management of the oral cavity, injury to the digestive tract, surgical operations, and the like. When bacteria enter the bloodstream, platelets can agglutinate and cause sepsis. Smaller platelets and fibrin are deposited on the endothelial surface of blood vessels and cause endocarditis [41]. Pathogenesis is related to the peptides and proteins produced by bacteria in the blood, which are thought to interfere with the binding sites of fibrinogen and fibronectin. This study was conducted to determine whether the *W. cibaria* strains induced platelet aggregation by measuring the platelet aggregation reaction, which is the initial stage of thrombin formation. The local reference range was 66–117 s for C/ADP and 86–164 s for C/EPI [42]. The result of the present study showed that *W. cibaria* strains had normal CT values as measured by C/EPI and C/ADP cartridges. This result showed that *W. cibaria* CMU and CMS1 strains did not induce platelet aggregation. To our knowledge, this study is the first to show that *W. cibaria* has no platelet aggregation ability.

Bourdichon et al. [43] created a list called the “Inventory of Microbial Food Cultures,” which included microbial species recorded as present in fermented foods. Conversely, species that are not considered as undesirable for food, those that do not have the metabolic activity of interest, and microbial species that lack data related to food fermentation were excluded from this list. The authors include *Weissella* spp. as useful microorganisms. However, *Weissella* spp. are not yet FDA-GRAS. *Weissella* spp. participate in the fermentation process with *Lactobacillus*, *Leuconostoc*, *Lactococcus*, and *Pediococcus*, but *Lactobacillus* spp. generally remain the dominant species; furthermore, *Lactobacillus* has been in use for centuries and has a GRAS status. *W. cibaria* was introduced for the first time in 2002 as a result of the development of molecular biology technology [44]. However, commercialization is not yet active, probably because it was not used as a starter, as it lacks the GRAS status. This has caused a reluctance to use GRAS for industrialization and a preference for LAB such as *Lactobacillus* or *Bifidobacterium*, which are recognized as GRAS.

*W. cibaria* strains are known to exert various beneficial effects, promoting their use as probiotics, such as anticancer effects, immunomodulatory effects, and anti-inflammatory effects in addition to dextran-producing as well as oral health effects [12,13,14,15,45,46,47,48]. On the other hand, Abriouel et al. [49] investigated the pathogenic potential of *Weissella* strains by analyzing the genome of *W. cibaria* KACC 11862 and identifying the virulence factor hemolysin and ARGs. However, in this study, *W. cibaria* CMU and CMS1 strains were found to be safe strains because they did not have ARGs, antibiotic resistance transferability, or virulence factors, indicating that the safety of this species was strain-specific. In the future, if these strains are GRAS approved, it is expected that they will be applied worldwide as a raw material for various types of oral health functional and fermented foods.

## 4. Materials and Methods

### 4.1. Bacterial Strains and Growth Conditions

Two *Weissella cibaria* strains including CMU (oraCMU) and CMS1 (oraCMS1), which have oral care probiotic properties, were investigated for safety evaluation (Oradentics Inc., Seoul, Korea) [14]. *Enterococcus faecalis* KCTC 3511 (ATCC 29212), *Enterococcus faecium* KCTC 13225 (ATCC 19434), *Lactobacillus rhamnosus* GG (KCTC 5033), *Listeria ivanovii* subsp. *ivanovii* KCTC 3444, *Proteus vulgaris* KCTC 2512, *Eubacterium limosum* KCTC 3266 (ATCC 8486), and *W. cibaria* KCTC 3807 were purchased from the Korean Culture Collection for Type Cultures (Daejeon, Korea). *Pseudomonas aeruginosa* KCCM 11328 (ATCC 27853) was purchased from the Korean Culture Center of Microorganisms (Seoul, Korea). *Escherichia coli* O157:H7 was provided by Chonnam National University (Gwangju, Korea). *Lactobacillus reuteri* (Stockholm, Sweden) and *Lactobacillus salivarius* (Tokyo, Japan) were also isolated from commercial oral probiotic products and identified through 16S rRNA sequence analysis. *W. cibaria*, *Lactobacillus* sp., and *E. faecium* were grown aerobically in De Man, Rogosa, and Sharpe broth (MRS broth, Difco, Detroit, MI, USA) at 37 °C for 16 h. *E. coli*, *E. faecalis*, *L. ivanovii*, *P. aeruginosa*, and *P. vulgaris* were grown aerobically in brain heart infusion broth (BHI broth, Difco) at 37 °C for 16 h. *E. limosum* was grown anaerobically (85% N_2_, 10% H_2_, and 5% CO_2_) in Reinforced Clostridial Medium (Difco) at 37 °C for 24 h.

### 4.2. Antibiotic Resistance

#### 4.2.1. Preparation of Antibiotics

All antibiotics (ampicillin, chloramphenicol, ciprofloxacin, clindamycin, erythromycin, fusidic acid, gentamicin, kanamycin, linezolid, oxytetracycline, rifampicin, streptomycin, tetracycline, and vancomycin) were purchased from Sigma (St. Louis, MO, USA). Each antibiotic was dissolved and filter sterilized prior to its addition to LSM broth medium, composed of 90% Iso-Sensitest broth (Kisan Bio Co., Ltd., Seoul, Korea) and 10% MRS broth. LSM broth medium was used as the diluent for subsequent two-fold serial dilutions in the microdilution method.

#### 4.2.2. Determination of Minimum Inhibitory Concentrations

The minimum inhibitory concentration (MIC) was determined using the microdilution method described by the International Organization of Standardization (ISO)/International Dairy Federation (IDF) [50]. Briefly, bacterial suspensions were adjusted until the optical density (OD) of the suspension at 600 nm was 0.5 and then diluted 10-fold. Microdilution plates were prepared with 100 μL of diluted inoculum and 100 μL of the series of two-fold dilutions of antibiotics. The plates were incubated at 37 °C for 48 h under anaerobic conditions. MIC values were read as the lowest concentration of an antimicrobial agent at which visible growth was inhibited. The assay was replicated three times. Strains showing MIC values less than EFSA’s breakpoints were considered sensitive; otherwise, they were recorded to be resistant [17].

#### 4.2.3. ARG Detection by PCR

Genomic DNA was extracted from the bacteria using a QIAamp DNA Mini Kit (Qiagen, Hilden, Germany). The extraction was performed according to the manufacturer’s instruction manuals. The concentration of the total bacterial DNA was determined using a MaestroNano spectrophotometer (Maestrogen, Las Vegas, NV, USA). Plasmid DNA was also extracted from the bacteria (OD_600_ of 0.6–0.8) using a QIAprep Spin Miniprep kit (Qiagen) according to the manufacturer’s instructions. Polymerase chain reactions (PCRs) were used to detect ARGs by gene-specific primers (Table 7). The amplification program was an initial denaturation step of 95 °C for 5 min, followed by 35 cycles of: 95 °C for 30 s, annealing temperature for 30 s, 72 °C for 1.5 min, and 72 °C for 7 min. The PCR products were subjected to 1% agarose gel electrophoresis followed by ethidium bromide staining. The resulting PCR products were purified with a MultiScreen filter plate (Millipore, Eschborn, Germany) and DNA sequenced with the BigDye Terminator v3.1 Cycle Sequencing kit (Applied Biosystems, Foster City, CA, USA) using the forward or reverse primer. Analysis of DNA sequences was performed with the BLAST program available at the Comprehensive Antibiotic Resistance Database (CARD) (https://card.mcmaster.ca/analyze/blast) [22].

#### 4.2.4. Antibiotic Resistance Transferability Test

The ability of donors to confer antibiotic resistance to the recipients was investigated. Conjugal transfer of antibiotic resistance was assessed by the filter mating method [63]. In brief, donor strains (*W. cibaria*) were cultured in MRS broth, supplemented with the specific antibiotic, and recipient strains (*E. faecalis* or *L. rhamnosus*) were grown in BHI or MRS broth without antibiotics. Two percent of the overnight culture bacteria were inoculated to the fresh media and incubated for 4 h to reach the midexponential phase (OD_600_ 0.2 to 0.5). Equal volumes (1 mL) of the donor and recipient bacteria were mixed and filtered through a sterile membrane filter (2.5 cm diameter, 0.45 µM; MF-Millipore membrane filter, HAWP02500; Millipore). To ensure cells attached tightly to the membrane, 10 mL of saline (0.85% NaCl) was passed through the filter after filtration, and the membrane was placed with the cell side facing upward on the surface of MRS agar and incubated at 37 °C for 24 h. The membrane was placed in 2 mL of saline, and the plate was washed again with 1 mL of saline. The wash was placed in a membrane-containing tube and mixed by vortexing to remove bacteria from the membrane. Dilutions were plated on selective agar for donors, recipients, and transconjugants for 48 h at 37 °C. Three replicates of all matings were conducted.

#### 4.2.5. Genome Analysis for ARGs

To determine whether ARGs were present in *W. cibaria*, WGS of the two strains of *W. cibaria* were used to identify the genetic variation. The analysis protocol was similar to the analysis protocol provided by ARDB (http://ardb.cbcb.umd.edu/index.html) [64], and the analysis proceeded without arbitrary adjustment values. CARD BLAST and RGI (https://card.mcmaster.ca/analyze/rgi) were also used for genome-based analysis of ARGs [22].

#### 4.2.6. Detection of Virulence Genes

The virulence genes in *W. cibaria* CMU, CMS1, *L. rhamnosus* GG, and *E. faecalis* ATCC 29212 were searched using the VirulenceFinder 2.0 Server, which is a component of the publicly available web-based tool for whole-genome sequencing (WGS) analysis provided by the Center for Genomic Epidemiology (CGE) (http://www.genomicepidemiology.org/). The database system was designed to detect homologous sequences for the virulence genes related to *E. coli*, *Listeria*, *S. aureus*, and *Enterococcus* in WGS data [65]. The output was composed of the best-matching genes in the BLAST analysis of selected databases for the submitted genomes of *W. cibaria* strains. The threshold for percent identity (%ID) and minimum length were set at 90% and 60%, respectively. If there was a matching result, the output showed the information about the predicted virulence gene, %ID, length of the query and database genes, the position of the hit in the contig, and the accession number of the hit.

### 4.3. Hemolytic Activity Test

Hemolytic activity was evaluated by plating and culturing bacteria on agar plates, which contained growth medium supplemented with 5% sheep blood. *W. cibaria* strains and commercial probiotic isolates or *L. ivanovii* as a positive control for hemolysis activity were aerobically cultivated by the streak plate method at 37 °C for 48 h [29]. Strains that displayed clear zones around the colonies were classified as microorganisms with β-hemolysis properties.

### 4.4. Mucin Degradation Test

Mucin degradation tests were performed according to previous reports, with slight modifications [30]. Briefly, bacteria were inoculated into 10 mL of MRS basal medium containing 0.3% partially purified mucin (type III, Sigma), with or without 1% glucose, and aerobically cultured at 37 °C for 24 h. Composition of the MRS basal medium was 10 g of enzyme digest casein, 10 g of beef extract, 5 g of sodium acetate, 4 g of yeast extract, 2 g of triammonium citrate, 2 g of K_2_HPO_4_, 1.08 g of Tween 80, 0.2 g of MgSO_4_, 0.05 g of MnSO_4_, and 1 L of distilled water. After incubation for 8 and 24 h, bacterial growth was assessed by measuring the absorbance at 600 nm (VersaMax, Molecular devices, San Jose, CA, USA) and pH of the culture.

### 4.5. Toxic Metabolite Production Test

#### 4.5.1. d-Lactic Acid Production Test

The production of d-lactic acid by *W. cibaria* strains was determined by culturing the bacteria in MRS medium for 24 h at 37 °C and quantifying the d-lactic acid in the medium using the d-lactate colorimetric assay kit from BioVision Research (Mountain View, CA, USA).

#### 4.5.2. Bile Salt Deconjugation Test

Bile salt deconjugation was analyzed by culturing *W. cibaria* strains for 24 h at 37 °C in MRS broth containing 1 mM taurocholic acid (TCA; Sigma) or glycocholic acid (GCA; Sigma). Deconjugation of the primary bile salts by *W. cibaria* strains was examined through HPLC (YL9100 Plus; YL Instruments Co., Anyang, Korea). HPLC with an Eclipse Plus C18 column (4.6 × 250 mm, 5 µm; Agilent Technologies, Santa Clara, CA, USA) and UV detector (Model M 720; YL) was used to analyze TCA and GCA. A 20 mL sample was injected into the column by Rheodyne injector (Rheodyne, L. P., Cotati, CA, USA), and the peak areas at 205 nm were calculated using Autochro-3000 software (YL). A mixture containing 700 mL of methanol and 300 mL of 20 mM acetic acid was used as a mobile phase. The pH of the solvent mixture was increased to 5.6 by addition of 5 M NaOH followed by filtration through a 0.45 µm nylon filter (Millipore). Sep-Paks C18 cartridges (Waters Associates, Milford, MA, USA) were used. The secondary bile acids such as deoxycholic acid (DCA) and lithocholic acid (LCA) were detected by TLC plate (Merck, Darmstadt, Germany). In brief, the bacterial culture was washed and suspended in 0.1 M sodium phosphate buffer, and 1 mL of 0.016% cholic acid (CA) and chenodeoxycholic acid (CDCA), the primary bile acids, were mixed and reacted at 37 °C for 30 min. Then, 6 N HCl was added (100 µL) to stop the enzymatic reaction. The fraction extracted by ethyl acetate was evaporated under nitrogen. The precipitates were analyzed by TLC. *E. limosum* was used as a positive control.

#### 4.5.3. Urease Activity Test

The activity of urease was detected by measuring the final pH of inoculated urea agar (growth medium supplemented with urea 20.0% and phenol red 0.012%, pH 6.9) [36]. *W. cibaria* strains were aerobically cultivated by the streak plate method at 37 °C for 24 h. *P. vulgaris* was used as a positive control. When the medium changed to pink or red, it was regarded as an ammonia-producing positive strain.

#### 4.5.4. β-Glucuronidase Activity Test

The activity of β-glucuronidase was detected by measuring the changed color using a 0.1 M *p*-nitrophenyl-β-d-glucuronide as a substrate [40]. The bacterial culture was concentrated and washed with sodium phosphate buffer (pH 7.0) until OD_600_ = 4. Substrates (200 µL) were added to an equal volume of bacterial sample and incubated at 37 °C for 16 h. When the medium changed to yellow, it was regarded as a positive strain. Additionally, 19 kinds of enzymatic activities including β-glucuronidase were evaluated by using the API ZYM test kit (bioMérieux, Marcy l’Etoile, France) as described by the manufacturer. The results were graded from zero (no activity) to five (>40 nanomoles) by comparing color intensity, and they were judged to be positive if the result was more than three (following the manufacturer’s instructions).

#### 4.5.5. Indole Production Test

Indole production was evaluated by tryptophan media [38]. Bacteria were grown in tryptophan media for 18 h at 37 °C. *E. coli* was used as a positive control. After incubation, five drops of Kovac’s reagent (10 g of *p*-dimethylaminobenzaldehyde, 150 mL of butanol, and 50 mL of hydrochloric acid) were added, and a positive bacterial strain in the tube was determined by a red color.

#### 4.5.6. Nitroreductase Activity Test

Nitroreductase activity was determined by the conversion of 4-nitrobenzoic acid to 4-aminobenzoic acid, and an assay was performed according to a previously described method [39]. In brief, a bacterial pellet obtained by centrifugation (3000× *g* for 10 min) was sonicated for 5 min. Subsequently, the supernatant was prepared by centrifugation. 4-Nitrobenzoic acid (30 mg/L) and trichloroacetic acid (0.21%) were added to the supernatant and incubated at 37 °C for 1 h. The amount of 4-aminobenzoic acid produced was detected by diazotization of the amine in the presence of sodium nitrite (0.007%) and addition of NEDD (*N*-(1-naphthyl ethylenediamine dihydrochloride) (0.35%) at 4 °C. When the medium produced a violet color, it was regarded as a positive reaction.

#### 4.5.7. Gelatin Liquefaction Test

A gelatin liquefaction assay was done in a tube containing nutrient gelatin (gelatin 12.0%, peptone 0.5%, and beef extract 0.3%). *W. cibaria* strains were cultured in MRS supplemented with nutrient gelatin. Bacteria were inoculated in the tubes and incubated at 37 °C for 24 h. *P. aeruginosa* was used as a positive control. When a liquefaction reaction occurred at 4 °C, it was regarded as a positive reaction [40].

#### 4.5.8. Phenylalanine Degradation Test

A phenylalanine degradation test was done in phenylalanine slant agar (NaCl 0.5%, yeast extract 0.3%, d,l-phenylalanine 0.2%, and Na_2_HPO_4_ 0.1%) [38]. *W. cibaria* strains were cultured in MRS agar supplemented with d,l-phenylalanine 0.2% for 24 h. *P. vulgaris* was used as a positive control. After incubation, five to ten drops of 10.0% ferric chloride were dropped on the slant agar. When the test tube turned green within 1 to 5 min, it was regarded as a positive strain.

### 4.6. Platelet Aggregation Test

The potential for *W. cibaria* strains to induce platelet aggregation was evaluated in whole blood specimens using a PFA-100 (Dade Behering, Marbug, Germany) [41]. In brief, bacterial cultures of *W. cibaria* were centrifuged, washed, and the pellet was obtained by saline (OD_600_ = 1). Blood (2.7 mL; 2.28 × 10^8^ platelets) and bacteria (50 µL; ~5 × 10^7^ CFU) or saline (50 µL) as a negative control were mixed. Mixed samples (900 µL) were applied to two disposable cartridges containing collagen/adenosine diphosphate (C/ADP) or with collagen/epinephrine (C/EPI), and then closure time (CT) was measured. Blood samples were obtained from volunteers who gave informed consent to participate in the study, which was carried out in accordance with the Declaration of Helsinki and under the terms of relevant local legislation.

## 5. Conclusions

*W. cibaria* CMU and CMS1 strains were found to be free of ARGs in both chromosomes and plasmids, and genetic analysis confirmed that antibiotic resistance to kanamycin and vancomycin was an intrinsic characteristic of *W. cibaria*. In addition, *W. cibaria* CMU as well as CMS1 appear to be safe because they lack antibiotic resistance transferability. Additionally, the genomic sequences of these *W. cibaria* strains did not include virulence genes related to pathogenic bacteria. Results of the β-hemolysis, mucin degradation, and platelet aggregation tests on *W. cibaria* strains showed negative reactions, further supporting the safety of these strains. From the results of toxic metabolic production, including d-lactic acid, bile salt deconjugation, ammonia production, β-glucuronidase activity, indole production, nitroreductase activity, phenylalanine degradation, and gelatin liquefaction, *W. cibaria* strains showed negative reactions. Therefore, our study confirms that the commercially available *W. cibaria* CMU and CMS1 strains are safe in accordance with international standards and can achieve the GRAS status in the future.

## Figures and Tables

**Table 1:** Antibiotic resistance profiles and minimum inhibitory concentration (MIC) values of the studied bacterial strains.

Antibiotics	EFSA Cut-Off (mg/L) ^a^					CMU	CMS1	Lr	Ls	Lrh	Ef
	*Lactobacillus* Obligate Heterofermentative	*L. reuteri*	*L. salivarius*	*L. rhamnosus*	*Enterococcus*						
AMP	4	2	4	4	4	0.5	0.5	0.5	0.5	1	1
VAN	N/R	N/R	N/R	N/R	4	>256	>256	256	>256	>256	2
GEN	16	8	16	16	32	16	4	8	8	8	256
KAN	32	64	64	64	512	128	32	128	128	32	256
STR	64	64	64	32	128	64	16	32	32	8	>256
ERY	1	1	1	1	4	0.03	0.03	0.25	0.03	0.03	2
CLIN	1	1	1	1	4	0.03	0.03	0.03	0.03	0.03	16
TET	8	16	8	8	2	8	4	4	1	0.25	32
CHL	4	4	4	4	8	4	4	4	2	1	4
FUS						32	16	8	2	256	4
OXY						8	4	4	0.5	0.1	8
RIF						16	8	8	0.5	0.1	2
CIP						2	2	4	1	0.25	1
LIN						2	1	2	0.5	0.5	2

^a^ European Food Safety Authority (EFSA) 2012. AMP, ampicillin; VAN, vancomycin; GEN, gentamicin; KAN, kanamycin; STR, streptomycin; ERY, erythromycin; CLIN, clindamycin; TET, tetracycline; CHL, chloramphenicol; FUS, fusidic acid; OXY, oxytetracycline; RIF, rifampicin; CIP, ciprofloxacin; and LIN, linezolid. N/R, not required. CMU, *W. cibaria* CMU; CMS1, *W. cibaria* CMS1; Lr, *L. reuteri* isolated from a commercial product (Sweden); Ls, *L. salivarius* isolated from a commercial product (Japan); Lrh, *L. rhamnosus* GG (KCTC 5033); and Ef, *E. faecalis* ATCC 29212.

**Table 2:** Antibiotic resistance genes (ARGs) of the studied bacterial strains.

Antibiotics	Target Genes	CMU	CMS1	Lr	Ls	Lrh	Ef
GEN	*aac*(6′)-*aph*(2”)	-	-	-	-	-	-
	*aac*(6′)*Ie-aph*(2”)*La*	-	-	-	-	-	-
CHL	*catA*	-	-	-	-	-	-
	*cat*	-	-	-	-	-	-
STR	*aadA*	-	-	-	-	-	-
	*aadE*	-	-	-	-	-	-
	*ant*(6)	-	-	-	-	-	-
	*dfrD*	-	-	-	-	-	-
VAN	*vanE*	-	-	-	-	-	-
	*vanX*	-	-	-	-	-	-
AMP	*blaZ*	-	-	-	-	-	-
	*bla*	-	-	-	-	-	-
	*mecA*	-	-	-	-	-	-
TET	*tet*(M)	-	-	-	-	-	+
	*tet*(K)	-	-	-	-	-	-
	*tet*(W)	-	-	-	-	-	-
RIF	*rpoB*	-	-	-	-	-	-
KAN	*aph(3”)-III*	-	-	-	-	-	-
	*ant(2”)-I*	-	-	-	-	-	-
	*aph(3”)-I*	-	-	-	-	-	-
CIP	*gyrA*	-	-	-	-	-	-
	*parC*	-	-	-	-	-	-
CLIN	*lnu*(A)	-	-	-	-	-	-
	*lnu*(B)	-	-	-	-	-	-
ERY	*erm*(B)	-	-	-	-	-	-
	*erm*(B)-*1*	-	-	-	-	-	-
	*erm*(C)	-	-	-	-	-	-
LIN	*cfr*	-	-	-	-	-	-
Conjugative transposon integrons	Tn*916*/Tn*1545*	-	-	-	-	-	+

GEN, gentamicin; CHL, chloramphenicol; STR, streptomycin; VAN, vancomycin; AMP, ampicillin; TET, tetracycline; RIF, rifampicin; KAN, kanamycin; CIP, ciprofloxacin; CLIN, clindamycin; ERY, erythromycin; and LIN, linezolid. CMU, *W. cibaria* CMU; CMS1, *W. cibaria* CMS1; Lr, *L. reuteri* isolated from a commercial product (Sweden); Ls, *L. salivarius* isolated from a commercial product (Japan); Lrh, *L. rhamnosus* GG (KCTC 5033); and Ef, *E. faecalis* ATCC 29212.

**Table 3:** Transferability of vancomycin and kanamycin resistance from donors (*W. cibaria*) to recipients (*E. faecalis* and *L. rhamnosus*) (CFU/mL).

Antibiotics	Donor	Recipients		Transconjugants		Transfer Frequency
	CMU	Ef	Lrh	CMU + Ef	CMU + Lrh	
Van 64	1.03 × 10^8^ ± 8.41 × 10^7^	0	-	-	-	-
Tet 16	0	3.70 × 10^8^ ± 3.68 × 10^8^	-	-	-	-
Van 64 + Tet 16	0	0	-	0	-	0
Kan 64	2.50 × 10^6^ ± 7.07 × 10^5^	-	0	-	-	-
Fus 64	0	-	2.15 × 10^9^ ± 7.07 × 10^6^	-	-	-
Kan 64 + Fus 64	0	-	0	-	0	0

Van 64, vancomycin (64 mg/L); Kan 64, kanamycin (64 mg/L); Tet 16, tetracycline (16 mg/L); or Fus 64, fusidic acid (64 mg/L) were included in the single- or double-selective agar medium. CMU, *W. cibaria* CMU; Ef, *E. faecalis* ATCC 29212; and Lrh, *L. rhamnosus* GG.

**Table 4:** The results of ARG analysis based on genome sequences data.

Name	Accession No.	RGI Criteria	Perfect Hits	Strict Hits	Loose Hits
*W. cibaria* CMU, complete genome	CP013936	Perfect, Strict, complete genes only	0	0	0
*W. cibaria* CMS1, complete genome	CP022606		0	0	0
*L. rhamnosus* GG, complete genome	013198		0	0	0
*E. faecalis* ATCC 29212, complete genome	CP008816		0	2	0
RGI Criteria *	**Strict**		**Strict**		
ARO	*dfrE*		*tet(W/N/W)*		
Detection Criteria	Protein homologous model		Protein homologous model		
AMR Gene family	Trimethoprim resistant dihydrofolate reductase dfr		Tetracycline-ribosomal protection protein		
Drug Class	Diaminopyrimidine antibiotic		Tetracycline antibiotic		
Resistance mechanism	Antibiotic target replacement		Antibiotic target protection		
% Identity of Matching Region	98.78		68.65		
% Length of Reference Sequence	100.00		100.00		

* The results of the RGI criteria for the complete genome of *E. faecalis* ATCC 29212 are presented. Perfect hits, 100% identical to the reference sequence along its entire length; strict hits, likely homologs of AMR genes; and loose hits, hits with weak similarity. ARO, antibiotic resistance ontology; AMR, antimicrobial resistance.

**Table 5:** Virulence gene analysis of *W. cibaria* strains, *L. rhamnosus* and *E. faecalis*.

**Virulence Genes**		**CMU**	**CMS1**	**Lrh**	**Ef**
Shiga-toxin genes for *E. coli*		-	-	-	-
Virulence genes for *E. coli*		-	-	-	-
Virulence genes for *Listeria*		-	-	-	-
Exoenzyme genes for *S. aureus*		-	-	-	-
Toxin genes for *S. aureus*		-	-	-	-
Hostimm genes for *S. aureus*		-	-	-	-
Virulence genes for *Enterococcus*		-	-	-	+
Virulence Factor *	**Identity**	**Query/Template Length**	**Position in Contig**	**Protein Function**	**Accession Number**
*ElrA*	99.68	2172/2172	1,447,067.. 1,449,238		CP003726.1
*SrtA*	100	735/735	1,813,853.. 1,814,587		CP003726.1
*ace*	97.76	1743/1743	157,931.. 159,673	Collagen adhesin precursor	AF260879.1
*cCF10*	99.88	828/828	2,112,855.. 2,113,662		CP002491.1
*cOB1*	100	819/819	1,298,659.. 1,299,477		CP002621.1
*cad*	100	930/930	2,022,216.. 2,023,145		CP003726.1
*camE*	99.6	501/501	406,237.. 406,737	Sex pheromone cAM373 precursor	AF435437.1
*ebpA*	99.97	3312/3312	147,276.. 150,587		CP002491.1
*ebpB*	99.86	1431/1431	150,591.. 152,021		CP002491.1
*efaAfs*	100	927/927	995,578.. 996,504		FP929058.1
*gelE*	99.87	1530/1530	824,819.. 826,348		CP002491.1
*hylA*	99.42	3266/3264	1,790,825.. 1,794,090		CP002491.1
*tpx*	100	510/510	1,704,855.. 1,705,364		CP002621.1

-, not detected; +, detected. CMU, *W. cibaria* CMU; CMS1, *W. cibaria* CMS1; Lrh, *L. rhamnosus* GG; and Ef, *E. faecalis* ATCC 29212. * The results of the virulence factor for the complete genome of *E. faecalis* ATCC 29212 are presented.

**Table 6:** Enzymatic profiles of the studied bacterial strains determined by API ZYM test which detects the activity of enzymes on 19 substrates.

Enzyme	CMU	CMS1	Lr	Ls	Lrh	Ef	Li
Alkaline phosphatase	-	-	-	-	-	-	-
Esterase (C4)	-	-	+	-	-	+	+
Esterase lipase (C8)	-	-	-	-	+	-	-
Lipase (C14)	-	-	-	-	-	-	-
Leucine arylamidase	-	-	+	+	+	+	-
Valine arylamidase	-	-	-	-	+	-	-
Cystine arylamidase	-	-	-	-	-	-	-
Trypsin	-	-	-	-	-	-	-
α-chymotrypsin	-	-	-	-	+	+	-
Acid phosphatase	+	+	+	+	+	+	+
Naphthol-AS-BI-phosphohydrolase	+	+	+	+	+	+	+
α-galactosidase	-	-	+	+	-	-	-
β-galactosidase	-	-	+	+	+	-	-
β-glucuronidase	-	-	-	-	-	-	-
α-glucosidase	-	-	+	-	+	-	-
β-glucosidase	-	-	-	-	+	-	+
*N*-acetyl-β-glucosaminidase	-	-	-	-	-	-	+
α-mannosidase	-	-	-	-	-	-	-
α-fucosidase	-	-	-	-	+	-	-

CMU, *W. cibaria* CMU; CMS1, *W. cibaria* CMS1; Lr, *L. reuteri* isolated from a commercial product (Sweden); Ls, *L. salivarius* isolated from a commercial product (Japan); Lrh, *L. rhamnosus* GG (KCTC 5033); Ef, *E. faecalis* ATCC 29212; and Li, *L. ivanovii* KCTC 3444.

**Table 7:** Gene-specific primers and conditions for polymerase chain reaction (PCR) detection.

Antibiotics	Target Genes	Oligo Sequences (5′–3′)	Annealing Temperature (°C)	Amplicon Size (bp)	References
GEN	*aac*(6′)-*aph*(2”)	CCAAGAGCAATAAGGGCATA	60	220	[51]
		CACTATCATAACCACTACCG			
	*aac*(6′)*Ie-aph*(2”)*La*	CAGAGCCTTGGGAAGATGAAG	58	348	[52]
		CCTCGTGTAATTCATGTTCTGGC			
CHL	*catA*	GGATATGAAATTTATCCCTC	50	486	[51]
		CAATCATCTACCCTATGAAT			
	*cat*	TTAGGTTATTGGGATAAGTTA	48	300	[53]
		GCATGRTAACCATCACAWAC			
STR	*aadA*	ATCCTTCGGCGCGATTTTG	56	282	[54]
		GCAGCGCAATGACATTCTTG			
	*aadE*	ATGGAATTATTCCCACCTGA	50	565	[54]
		TCAAAACCCCTATTAAAGCC			
	*ant*(6)	ACTGGCTTAATCAATTTGGG	53	597	[55]
		GCCTTTCCGCCACCTCACCG			
VAN	*vanE*	TGTGGTATCGGAGCTGCAG	52	513	[56]
		GTCGATTCTCGCTAATCC			
	*vanX*	TCGCGGTAGTCCCACCATTCGTT	55	454	[57]
		AAATCATCGTTGACCTGCGTTAT			
AMP	*blaZ*	ACTTCAACACCTGCTGCTTTC	58	240	[58]
		TAGGTTCAGATTGGCCCTTAG			
	*bla*	CATARTTCCGATAATASMGCC	51	297	[53]
		CGTSTTTAACTAAGTATSGY			
	*mecA*	GGGATCATAGCGTCATTATTC	58	1429	[58]
		AGTTCTGCAGTACCGGATTTGC			
TET	*tet*(M)	GGTGAACATCATAGACACGC	55	401	[59]
		CTTGTTCGAGTTCCAATGC			
	*tet*(K)	TCGATAGGAACAGCAGTA	55	169	[58]
		CAGCAGATCCTACTCCTT			
	*tet*(W)	GAGAGCCTGCTATATGCCAGC	64	168	[56]
		GGGCGTATCCACAATGTTAAC			
RIF	*rpoB*	TAACCGTGGTGCTTGGCTDGAATWYGAAAC	59	1100	[60]
		ATCAAACCAATGTTAGGNCCTTCWGGDGTTTC			
KAN	*aph* (3”)-*III*	GCCGATGTGGATTGCGAAAA	52	292	[54]
		GCTTGATCCCCAGTAAGTCA			
	*ant*(2”)-*I*	GGGCGCGTCATGGAGGAGTT	67	329	[54]
		TATCGCGACCTGAAAGCGGC			
	*aph*(3”)-*I*	AACGTCTTGCTCGAGGCCGCG	68	670	[54]
		GGCAAGATCCTGGTATCGGTCTGCG			
CIP	*gyrA*	GAYTATGCWATGTCAGTTATTGT	45	286	[54]
		GGAATRTTRGAYGTCATACCAAC			
	*parC*	TATTCYAAATAYATCATTCARGA	50	286	[53]
		GCYTCNGTATAACGCATMGCCG			
CLIN	*lnu*(A)	GGTGGCTGGGGGGTAGATGTATTAACTGG	55	323	[56]
		GCTTCTTTTGAAATACATGGTATTTTTCGATC			
	*lnu*(B)	CCTACCTATTGTTTGTGGAA	54	925	[57]
		ATAACGTTACTCTCCTATTTC			
ERY	*erm*(B)	GAAAAGGTACTCAACCAAATA	54	639	[58]
		AGTAACGGTACTTAAATTGTTTAC			
	*erm*(B)-*1*	CATTTAACGACGAAACTGGC	54	405	[55,59]
		GGAACATCTGTGGTATGGCG			
	*erm*(C)	TCAAAACATAATATAGATAAA	50	642	[58]
		GCTAATATTGTTTAAATCGTCAAT			
LIN	*cfr*	TGAAGTATAAAGCAGGTTGGGAGTCA	55	746	[61]
		ACCATATAATTGACCACAAGCAGC			
Conjugative transposon	*int* (Tn*916*/Tn*1545*)	GCGTGATTGTATCTCACT	55	1046	[62]
		GACGCTCCTGTTGCTTCT			

GEN, gentamicin; CHL, chloramphenicol; STR, streptomycin; VAN, vancomycin; AMP, ampicillin; TET, tetracycline; RIF, rifampicin; KAN, kanamycin; CIP, ciprofloxacin; CLIN, clindamycin; ERY, erythromycin; and LIN, linezolid.

## References

[1] Lilly D.M., Stillwell R.H. (1965). Probiotics: Growth-promoting factors produced by microorganisms. Science.

[2] Behnsen J., Deriu E., Sassone-Corsi M., Raffatellu M. (2013). Probiotics: Properties, examples, and specific applications. Cold Spring Harb. Perspect. Med..

[3] Global Market Insights Inc. Probiotics Market Size to Exceed USD 64 Billion by 2023: Global Market Insights Inc.. https://www.prnewswire.com/news-releases/the-global-probiotics-market-size-is-expected-to-reach-usd-66-03-billion-by-2024--300726946.html.

[4] Ku S., Park M.S., Ji G.E., You H.J. (2016). Review on *bifidobacterium bifidum* BGN4: Functionality and nutraceutical applications as a probiotic microorganism. Int. J. Mol. Sci..

[5] Food and Agriculture Organization-World Health Organization (FAO/WHO) (2002). Report on Joint FAO/WHO Guidelines for the Evaluation of Probiotics in Food. http://www.who.int/foodsafety/fsmanagement/en/probiotic_guidelines.pdf.

[6] Dewhirst F.E., Chen T., Izard J., Paster B.J., Tanner A.C., Yu W.H., Lakshmanan A., Wade W.G. (2010). The human oral microbiome. J. Bacteriol..

[7] Romani Vestman N., Chen T., Lif Holgerson P., Öhman C., Johansson I. (2015). Oral microbiota shift after 12-week supplementation with *lactobacillus reuteri* DSM 17938 and PTA 5289; a randomized control trial. PLoS ONE.

[8] Marchetti E., Tecco S., Santonico M., Vernile C., Ciciarelli D., Tarantino E., Marzo G., Pennazza G. (2015). Multi-sensor approach for the monitoring of halitosis treatment via *lactobacillus brevis* (CD2)-containing lozenges—A randomized, double-blind placebo-controlled clinical trial. Sensors.

[9] Suzuki N., Yoneda M., Tanabe K., Fujimoto A., Iha K., Seno K., Yamada K., Iwamoto T., Masuo Y., Hirofuji T. (2014). *Lactobacillus salivarius* WB21—Containing tablets for the treatment of oral malodor: A double-blind, randomized, placebo-controlled crossover trial. Oral Surg. Oral Med. Oral Pathol. Oral Radiol..

[10] Mahasneh S.A., Mahasneh A.M. (2017). Probiotics: A promising role in dental health. Dent. J..

[11] Allaker R.P., Stephen A.S. (2017). Use of probiotics and oral health. Curr. Oral Health Rep..

[12] Kang M.S., Chung J., Kim S.M., Yang K.H., Oh J.S. (2006). Effect of *Weissella cibaria* isolates on the formation of *Streptococcus mutans* biofilm. Caries Res..

[13] Kang M.S., Kim B.G., Chung J., Lee H.C., Oh J.S. (2006). Inhibitory effect of *Weissella cibaria* isolates on the production of volatile sulphur compounds. J. Clin. Periodontol..

[14] Do K.H., Park H.E., Kang M.S., Kim J.T., Yeu J.E., Lee W.K. (2019). Effects of *Weissella cibaria* CMU on Halitosis and Calculus, Plaque, and Gingivitis Indices in Beagles. J. Vet. Dent..

[15] Lim H.S., Yeu J.E., Hong S.P., Kang M.S. (2018). Characterization of antibacterial cell-free supernatant from oral care probiotic *Weissella cibaria*, CMU. Molecules.

[16] List of Raw Materials Available for Food. https://www.foodsafetykorea.go.kr/foodcode/01_03.jsp?idx=12135.

[17] EFSA Panel on Additives and Products of Substances used in Animal Feed (FEEDAP) (2012). Guidance on the assessment of bacterial susceptibility to antimicrobials of human and veterinary importance. EFSA J..

[18] Woodford N., Ellington M.J. (2007). The emergence of antibiotic resistance by mutation. Clin. Microbiol. Infect..

[19] Mathur S., Singh R. (2005). Antibiotic resistance in food lactic acid bacteria—A review. Int. J. Food Microbiol..

[20] Choi A.R., Patra J.K., Kim W., Kang S.S. (2018). Antagonistic activities and probiotic potential of lactic acid bacteria derived from a plant-based fermented food. Front. Microbiol..

[21] Wang J., Wei X., Fan M. (2018). Assessment of antibiotic susceptibility within lactic acid bacteria and coagulase-negative staphylococci isolated from hunan smoked pork, a naturally fermented meat product in China. J. Food Sci..

[22] Jia B., Raphenya A.R., Alcock B., Waglechner N., Guo P., Tsang K.K., Lago B.A., Dave B.M., Pereira S., Sharma A.N. (2017). CARD 2017: Expansion and model-centric curation of the comprehensive antibiotic resistance database. Nucleic Acids Res..

[23] Mardassi B.B., Aissani N., Moalla I., Dhahri D., Dridi A., Mlik B. (2012). Evidence for the predominance of a single *tet*(M) gene sequence type in tetracycline-resistant *Ureaplasma parvum* and *Mycoplasma hominis* isolates from tunisian patients. J. Med. Microbiol..

[24] Bahl M.I., Sørensen S.J., Hansen L.H., Licht T.R. (2004). Effect of tetracycline on transfer and establishment of the tetracycline-inducible conjugative transposon Tn*916* in the guts of gnotobiotic rats. Appl. Environ. Microbiol..

[25] Scornec H., Bellanger X., Guilloteau H., Groshenry G., Merlin C. (2017). Inducibility of Tn*916* conjugative transfer in *Enterococcus faecalis* by subinhibitory concentrations of ribosome-targeting antibiotics. J. Antimicrob. Chemother..

[26] Giraffa G. (2003). Functionality of enterococci in dairy products. Int. J. Food Microbiol..

[27] Olawale K.O., Fadiora S.O., Taiwo S.S. (2011). Prevalence of hospital-acquired enterococci infections in two primary-care hospitals in Osogbo, Southwestern Nigeria. Afr. J. Infect. Dis..

[28] Franz C.M., Huch M., Abriouel H., Holzapfel W., Gálvez A. (2011). Enterococci as probiotics and their implications in food safety. Int. J. Food Microbiol..

[29] Gottschalk M.G., Lacouture S., Dubreuil J.D. (1995). Characterization of *Streptococcus suis* capsular type 2 haemolysin. Microbiology.

[30] Prakash R., Bharathi Raja S., Devaraj H., Devaraj S.N. (2011). Up-Regulation of MUC2 and IL-1β expression in human colonic epithelial cells by *Shigella* and its interaction with mucins. PLoS ONE.

[31] Domellöf L., Reddy B.S., Weisburger J.H. (1980). Microflora and deconjugation of bile acids in alkaline reflux after partial gastrectomy. Am. J. Surg..

[32] Begley M., Hill C., Gahan C.G. (2006). Bile salt hydrolase activity in probiotics. Appl. Environ. Microbiol..

[33] Muñoz-Atienza E., Gómez-Sala B., Araújo C., Campanero C., del Campo R., Hernández P.E., Herranz C., Cintas L.M. (2013). Antimicrobial activity, antibiotic susceptibility and virulence factors of lactic acid bacteria of aquatic origin intended for use as probiotics in aquaculture. BMC Microbiol..

[34] Adeva M., González-Lucán M., Seco M., Donapetry C. (2013). Enzymes involved in l-lactate metabolism in humans. Mitochondrion.

[35] Vitetta L., Coulson S., Thomsen M., Nguyen T., Hall S. (2017). Probiotics, D-Lactic acidosis, oxidative stress and strain specificity. Gut Microbes.

[36] Isenberg H.D. (1992). Urease. Clinical Microbiology Procedures Handbook.

[37] Heavey P.M., Rowland I.R. (2004). Microbial-gut interactions in health and disease. Gastrointestinal cancer. Best Pract. Res. Clin. Gastroenterol..

[38] Watanabe T., Snell E.E. (1972). Reversibility of the Tryptophanase reaction: Synthesis of tryptophan from indole, pyruvate, and ammonia. Proc. Natl. Acad. Sci. USA.

[39] Rafil F., Franklin W., Heflich R.H., Cerniglia C.E. (1991). Reduction of nitroaromatic compounds by anaerobic bacteria isolated from the human gastrointestinal tract. Appl. Environ. Microbiol..

[40] Zemelman R., Longeri L. (1965). Characterization of staphylococci isolated from raw milk. Appl. Microbiol..

[41] Korpela R., Moilanen E., Saxelin M., Vapaatalo H. (1997). *Lactobacillus rhamnosus* GG (ATCC 53103) and platelet aggregation in vitro. Int. J. Food Microbiol..

[42] Favaloro E.J. (2008). Clinical utility of the PFA-100. Semin. Thromb. Hemost..

[43] Bourdichon F., Casaregola S., Farrokh C., Frisvad J.C., Gerds M.L., Hammes W.P., Harnett J., Huys G., Laulund S., Ouwehand A. (2012). Food fermentations: Microorganisms with technological beneficial use. Int. J. Food Microbiol..

[44] Björkroth K.J., Schillinger U., Geisen R., Weiss N., Hoste B., Holzapfel W.H., Korkeala H.J., Vandamme P. (2002). Taxonomic study of *Weissella confusa* and description of *Weissella cibaria* sp. nov., detected in food and clinical samples. Int. J. Syst. Evol. Microbiol..

[45] Kwak S.H., Cho Y.M., Noh G.M., Om A.S. (2014). Cancer preventive potential of kimchi lactic acid bacteria (*Weissella cibaria, Lactobacillus plantarum*). J. Cancer Prev..

[46] Lee H.A., Song B.R., Kim H.R., Kim J.E., Yun W.B., Park J.J., Lee M.L., Choi J.Y., Lee H.S., Hwang D.Y. (2017). Butanol extracts of *Asparagus cochinchinensis* fermented with *Weissella cibaria* inhibit iNOS-mediated COX-2 induction pathway and inflammatory cytokines in LPS-stimulated RAW264.7 macrophage cells. Exp. Ther. Med..

[47] Hong Y.F., Lee Y.D., Park J.Y., Kim S., Lee Y.W., Jeon B., Jagdish D., Kim H., Chung D.K. (2016). Lipoteichoic acid isolated from *Weissella cibaria* increases cytokine production in human monocyte-like THP-1 cells and mouse splenocytes. J. Microbiol. Biotechnol..

[48] Baruah R., Maina N.H., Katina K., Juvonen R., Goyal A. (2017). Functional food applications of dextran from *Weissella cibaria* RBA12 from pummelo (Citrus maxima). Int. J. Food Microbiol..

[49] Abriouel H., Lerma L.L., Casado Muñoz Mdel C., Montoro B.P., Kabisch J., Pichner R., Cho G.S., Neve H., Fusco V., Franz C.M. (2015). The controversial nature of the *Weissella* genus: Technological and functional aspects versus whole genome analysis-based pathogenic potential for their application in food and health. Front. Microbiol..

[50] International Organization for Standardization (ISO) (2010). Milk and Milk Products-Determination of the Minimal Inhibitory Concentration (MIC) of Antibiotics Applicable to Bifidobacteria and Non-Enterococcal Lactic Acid Bacteria (LAB).

[51] Rojo-Bezares B., Sáenz Y., Poeta P., Zarazaga M., Ruiz-Larrea F., Torres C. (2006). Assessment of antibiotic susceptibility within lactic acid bacteria strains isolated from wine. Int. J. Food Microbiol..

[52] Bujnakova D., Strakova E., Kmet V. (2014). In vitro evaluation of the safety and probiotic properties of lactobacilli isolated from chicken and calves. Anaerobe.

[53] Hummel A.S., Hertel C., Holzapfel W.H., Franz C.M. (2007). Antibiotic resistances of starter and probiotic strains of lactic acid bacteria. Appl. Environ. Microbiol..

[54] Ouoba L.I., Lei V., Jensen L.B. (2008). Resistance of potential probiotic lactic acid bacteria and bifidobacteria of african and european origin to antimicrobials: Determination and transferability of the resistance genes to other bacteria. Int. J. Food Microbiol..

[55] Zhou N., Zhang J.X., Fan M.T., Wang J., Guo G., Wei X.Y. (2012). Antibiotic resistance of lactic acid bacteria isolated from chinese yogurts. J. Dairy Sci..

[56] Kastner S., Perreten V., Bleuler H., Hugenschmidt G., Lacroix C., Meile L. (2006). Antibiotic susceptibility patterns and resistance genes of starter cultures and probiotic bacteria used in food. Syst. Appl. Microbiol..

[57] Liu C., Zhang Z.Y., Dong K., Yuan J.P., Guo X.K. (2009). Antibiotic resistance of probiotic strains of lactic acid bacteria isolated from marketed foods and drugs. Biomed. Environ. Sci..

[58] Aquilanti L., Garofalo C., Osimani A., Silvestri G., Vignaroli C., Clementi F. (2007). Isolation and molecular characterization of antibiotic-resistant lactic acid bacteria from poultry and swine meat products. J. Food Prot..

[59] Gad G.F., Abdel-Hamid A.M., Farag Z.S. (2014). Antibiotic resistance in lactic acid bacteria isolated from some pharmaceutical and dairy products. Braz. J. Microbiol..

[60] Guo H., Pan L., Li L., Lu J., Kwok L., Menghe B., Zhang H., Zhang W. (2017). characterization of antibiotic resistance genes from *Lactobacillus* isolated from traditional dairy products. J. Food Sci..

[61] Morales G., Picazo J.J., Baos E., Candel F.J., Arribi A., Peláez B., Andrade R., de la Torre M.A., Fereres J., Sánchez-García M. (2010). Resistance to linezolid is mediated by the *cfr* Gene in the first report of an outbreak of linezolid-resistant *Staphylococcus aureus*. Clin. Infect. Dis..

[62] Macovei L., Zurek L. (2006). Ecology of antibiotic resistance genes: Characterization of enterococci from houseflies collected in food settings. Appl. Environ. Microbiol..

[63] Tannock G.W. (1987). Conjugal transfer of plasmid pAM β1 in *Lactobacillus reuteri* and between lactobacilli and *Enterococcus faecalis*. Appl. Environ. Microbiol..

[64] Liu B., Pop M. (2009). ARDB—Antibiotic Resistance Genes Database. Nucleic Acids Res..

[65] Joensen K.G., Scheutz F., Lund O., Hasman H., Kaas R.S., Nielsen E.M., Aarestrup F.M. (2014). Real-time whole-genome sequencing for routine typing, surveillance, and outbreak detection of verotoxigenic *Escherichia coli*. J. Clin. Micobiol..

